# Primary malignant melanoma of the vagina: A case report of a rare disease that is difficult to diagnose

**DOI:** 10.1097/MD.0000000000041259

**Published:** 2025-01-10

**Authors:** Soyoung Park, Jung-Woo Park, Seo-Hee Rha, Sujin Kim

**Affiliations:** a Department of Obstetrics and Gynecology, Dong-A University, College of Medicine, Busan, Republic of Korea; b Department of Pathology, Dong-A University College of Medicine, Busan, Republic of Korea.

**Keywords:** case report, malignant melanoma, postmenopause, vagina

## Abstract

**Rationale:**

Malignant melanoma is a rare cancer that accounts for approximately 1% of all cancers. Primary malignant melanoma of the female genital tract accounts for approximately 3% to 7% of all malignant melanomas, and 0.3% to 0.8% of all melanomas in women. It affects postmenopausal women ages 60 to 80 years. Various hormonal factors, including puberty, pregnancy, menopause, oral contraceptive use, and human papillomavirus infection are associated with primary malignant melanoma of the vagina.

**Patient concerns:**

Symptoms often include vaginal bleeding, discharge, and pain; however, it can also present as pigmented or nonpigmented lesions, making diagnosis challenging.

**Diagnoses:**

Diagnosis involves detailed history, physical examination, and imaging (CT, MRI, and positron emission tomography). Immunohistochemical staining for markers, such as human melanoma black-45 and Melan-A, is crucial for confirmation. The diagnosis was made through careful physical examination, imaging studies, and immunohistochemistry.

**Interventions:**

The treatment includes wide local excision, radical surgery, radiotherapy, chemotherapy, and immunotherapy. The prognosis of primary malignant melanoma of the vagina is usually poor owing to late diagnosis, and the 5-year survival rate is 5% to 25%.

**Outcomes and lessons:**

To consider the possibility of primary malignant melanoma of vagina, postmenopausal women, particularly those who with human papillomavirus infection, should be performed thorough examination regardless of symptoms of vaginal bleeding or discharge.

## 1. Introduction

Malignant melanoma, which constitutes approximately 1% of all cancers, is a rare type of cancer.^[1]^ It may involve any part of the body including the skin, oral cavity, and vulva. Primary malignant melanoma of the female genital tract represents approximately 3% to 7% of all malignant melanomas and 0.3% to 0.8% of all melanomas in women.^[1,2]^ Owing to delayed diagnosis, the prognosis for primary malignant melanoma of the vagina (PMMV) is generally unfavorable, with a 5-year survival rate of 5% to 25%.^[1,3]^

Patients with PMMV typically present with vaginal bleeding, vaginal discharge, or a palpable vaginal mass.^[4]^ The diagnosis was made through careful physical examination, imaging studies, and immunohistochemistry. Treatment includes wide local excision, radical surgery, chemotherapy, immunotherapy, and radiotherapy.^[5]^ This report presents the case of a 53-year-old female patient with PMMV and the clinical and histological features of the disease.

## 2. Case report

A 53-year-old female patient attended a regular outpatient clinic follow-up at the Department of Obstetrics and Gynecology of Dong-A University Hospital for an abnormal Pap smear result of atypical squamous cells of undetermined significance positive for human papillomavirus (HPV) (genotypes 39, 68, and 74). A punch biopsy was performed, and a low-grade squamous intraepithelial lesion (LSIL) was confirmed. 12 months later, a repeat Pap smear indicated LSIL and the patient underwent diagnostic conization. The pathological diagnosis was LSIL. A few months later, the follow-up Pap smear result indicated LSIL and was positive for HPV (genotypes 52 and 58). Colposcopy revealed an acetowhite epithelial lesion.

Six months later, the follow-up Pap smear result was atypical squamous cells-cannot exclude high-grade squamous intraepithelial lesion, and was positive for HPV (genotype 51). While performing the Pap smear, routine inspection revealed a brown pigmentation of the vagina. A punch biopsy of the pigmented vaginal lesion was performed, and atypical cells with brown pigmentation in the subepithelial connective tissue were confirmed. Specific immunohistochemical staining revealed a few cells that were positive for human melanoma black 45 (HMB-45) and Melan-A. Inconclusive pathological results prevented the diagnosis of malignant melanoma of the vagina; therefore, we delayed further assessment of the lesion until the next follow-up. After 3 months, the follow-up Pap smear indicated atypical squamous cells-cannot exclude high-grade squamous intraepithelial lesion and was positive for HPV (genotypes 50 and 51). Vaginal pigmentation was again detected on physical examination. A repeat punch biopsy was performed to examine the atypical cells in the subepithelial tissue.

**Figure 1.**
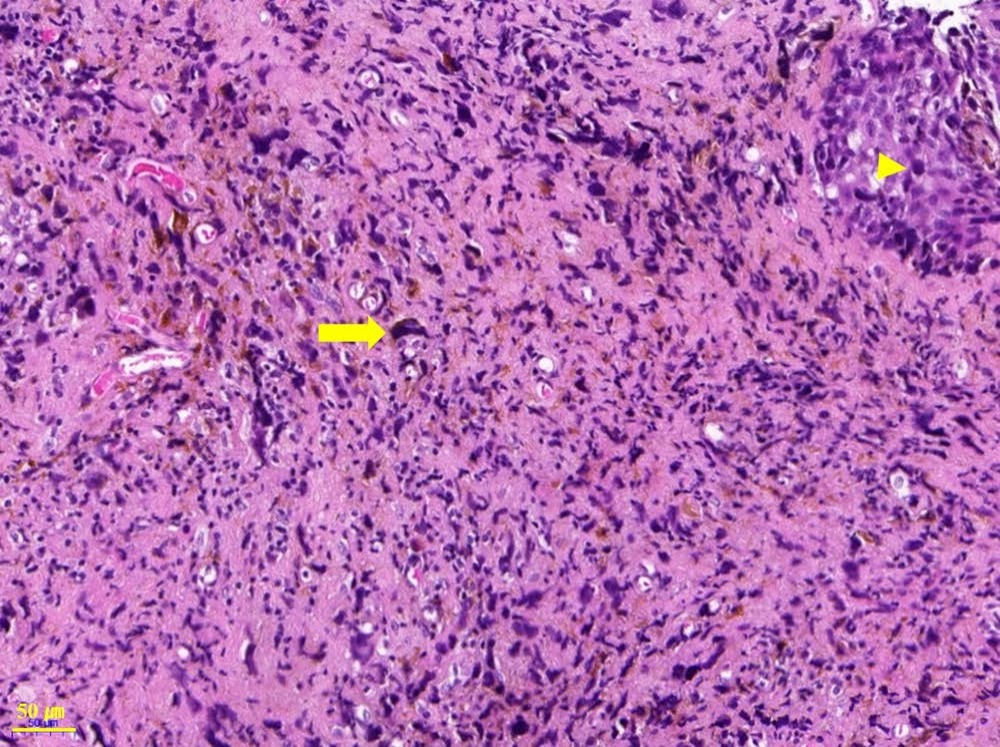
The vaginal mucosa showing numerous scattered atypical cells with brown pigment in the epithelium (arrowhead) and stroma (arrow).

**Figure 2.**
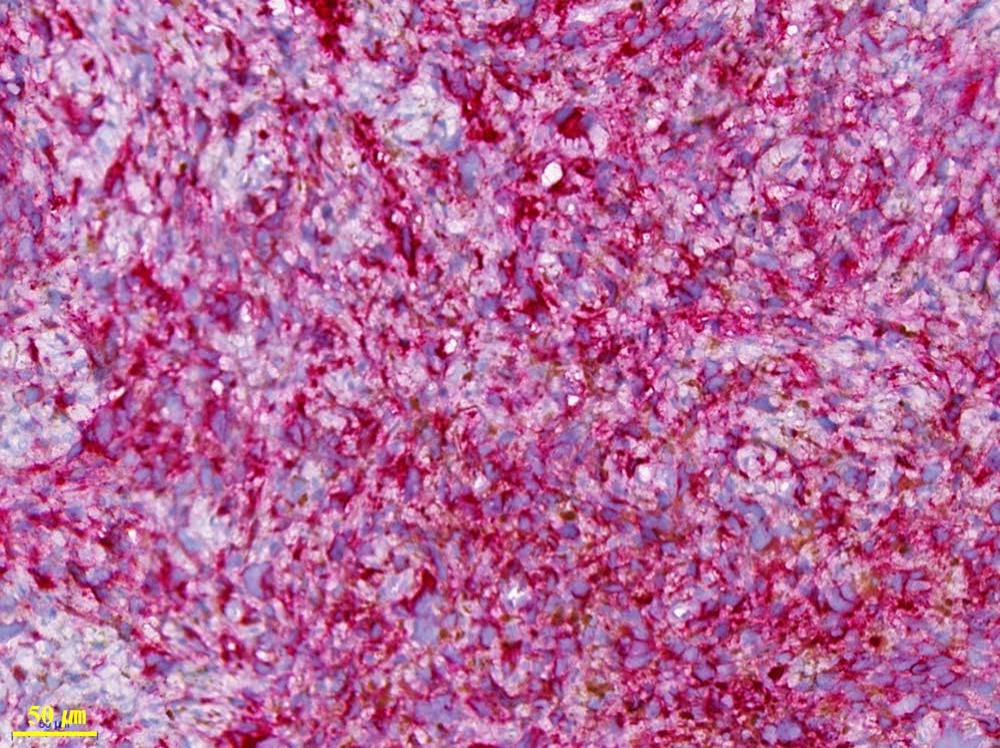
Atypical cells positive for the melanoma marker HMB-45 (red). HMB-45 = human melanoma black 45.

The patient underwent conization and vaginal wall excision because of persistent abnormal Pap smear results and brown pigmentation of the vaginal wall. The pathological diagnosis of cervical conization revealed chronic cervicitis, while the pathological diagnosis of vaginal wall was initially LSIL with an atypical subepithelial melanocytic lesion (Fig. 1). Specific immunohistochemical staining revealed positive results for melan-A, HMB-45, and Ki-67 (Fig. 2). Following thorough histological examination, the pathological diagnosis was changed to malignant melanoma with dermal invasion. Imaging for cancer evaluation revealed no distant metastasis. The patient underwent a total laparoscopic hysterectomy with bilateral salpingo-oophorectomy and total vaginectomy. The resection margin of the vagina was clear. One year after initial diagnosis and surgery, no signs of disease recurrence were observed.

## 3. Discussion

PMMV is rare. Its annual incidence is estimated to be 0.46 per 1 million women.^[5]^ It is often found in women aged 60 to 80 years, and most patients are menopausal.^[5]^

Melanocytes are derived from the embryonic neural crest, and subsequently migrate to the epidermis. During migration, a few melanocytes remain in the vaginal mucosa or endocervical canal.^[6]^ PMMV originates from melanocytes in the basal layer of the vaginal epithelium.^[2]^ It can be found in any part of the vagina; however, it is mostly found in the anterior wall of the lower third.^[1]^ Although our patient was 53 years of age at the time of diagnosis, she was menopausal.

The mechanisms underlying the pathogenesis and etiological factors of PMMV remain unknown.^[5]^ Socioeconomic factors such as education level, income, and poverty are unrelated to their occurrence.^[5]^ PMMV is associated with a KIT gene mutation, whereas cutaneous melanomas are often associated with BRAF gene mutation.^[5]^ In addition, hormonal influences such as puberty, pregnancy, menopause, oral contraceptive use, and HPV infection are associated with PMMV.^[6]^ Our patient showed continuous abnormal Pap smear results and positive HPV test results.

Patients typically present with vaginal bleeding, vaginal discharge, a palpable mass in the vagina, and pain.^[4]^ Cancer can be polypoid, nodular, or ulcerated.^[2]^ It is typically pigmented; however, in approximately 10% to 23% of cases, the cancer is nonpigmented.^[1]^ The latter can be difficult to detect, leading to misdiagnosis. Nonpigmented PMMV are similar to vaginal epithelial tumors.^[2]^ Differential diagnosis should be considered, including poorly differentiated squamous cell carcinoma, sarcoma, lymphoma, and blue nevus.^[2,7]^ Our patient had no symptoms of vaginal bleeding, vaginal discharge, or a palpable vaginal mass; however, a brown-pigmented lesion was detected on the vaginal wall.

The initial diagnosis can be confirmed by tissue biopsy, based on through history taking and physical examination. After diagnosis, additional imaging examinations, including computed tomography, magnetic resonance imaging, and positron emission tomography can be done. Hysteroscopy can play a crucial role in the contemporary study of the vagina, cervix, and endocervix, particularly in cases involving rare malignancies such as primary malignant melanoma of the cervix. This approach allowed for direct visualization and precise sampling of the lesion, which cytology and routine gynecological examinations might miss.^[8]^

In this case, PMMV was confirmed by immunohistochemical staining. Positive staining results for proteins S-100, Melan-A, HMB-45, and vimentin suggest PMMV.^[4]^ In our case, the tumor cells were positive for HMB-45 and Melan-A but negative for S-100.

The International Federation of Gynecology and Obstetrics and American Joint Committee on Cancer tumor, node, metastasis staging systems can be used for staging PMMV.^[5]^ Several studies have recommended American Joint Committee on Cancer-tumor, node, metastasis staging because of its detailed staging system and prognosis prediction.^[5,9]^

Surgery, with or without adjuvant therapy, is the preferred PMMV treatment.^[5]^ Treatment considerations should include wide local excision in the early stages of the tumor and radical surgery and lymphadenectomy with adjuvant chemotherapy or radiotherapy in advanced stages.^[5]^ Radical surgery can include total hysterectomy, total vaginectomy, vulvectomy, and pelvic exenteration, depending on tumor location.^[5,10]^ Lymphadenectomy is contraindicated in patients without lymph node metastasis.^[5]^ In inoperable advanced tumor stages, chemotherapy with dacarbazine, temozolomide, and paclitaxel should be considered for palliation.^[5]^ Interferon alpha-2b can be used as a postoperative adjuvant immunotherapy to prevent tumor relapse.^[5]^ Radiotherapy alone is not recommended for tumor treatment^[5]^; however, it can be used as a preoperative neoadjuvant treatment to decrease tumor size, postoperative adjuvant treatment for incomplete tumor resection, or pelvic metastasis.^[10]^ Furthermore, radiotherapy can be used as adjuvant treatment in patients without the option for surgery.

Immunotherapy is a new treatment strategy for PMMV. Recent studies have shown that immunotherapy with checkpoint inhibitors and targeted therapy improves the prognosis.^[1]^ Nivolumab, a programmed death-1 receptor monoclonal antibody, can improve overall and progression-free survival compared to chemotherapy.^[1]^ Therefore, immune checkpoint inhibitors or targeted therapies are recommended as first-line treatments for metastatic or inoperable PMMV in the presence of driver mutations.^[3]^ The recommended initial regimen is a combination of nivolumab and ipilimumab, or anti-PD1 monotherapy with pembrolizumab or nivolumab.^[3]^

PMMV is aggressive. Owing to delayed diagnosis and rich blood and lymphatic flow to the vaginal mucosa, many patients are diagnosed at an advanced tumor stage.^[10]^ These factors are also associated with early tumor spread and metastasis.^[10]^ PMMV prognosis is usually poor due to late diagnosis.^[7,10]^ In some studies, tumor size was one of the most important prognostic factors.^[4]^ A tumor size <3 cm is associated with an approximate survival time of 41 months and a better prognosis; in contrast, tumors > 3 cm are associated with an approximate survival time of 12 months.^[5]^ Other potential prognostic factors include age, International Federation of Gynecology and Obstetrics stage, tumor location, depth of invasion, pigmentation, ulceration, histology, cell type, number of mitoses, vascular invasion, type of surgical procedure, adjuvant radiotherapy, and chemotherapy.^[5]^ Lymph node metastasis significantly worsens the prognosis.^[9]^ Patients who undergo surgical treatment typically have a longer survival time than those who undergo nonsurgical treatments.^[5]^

## 4. Conclusion

Herein, we report a case of PMMV. This tumor is rare and has a poor prognosis due to late diagnosis. PMMV is typically pigmented; however, in some cases, it is nonpigmented and can be difficult to diagnose. Postmenopausal women may visit clinics for genitourinary syndrome of menopause, which has various menopausal signs and symptoms, including genital dryness, burning sensation, irritation, sexual discomfort, impairment, and urinary symptoms.^[11]^ Considering the possibility of PMMV, a thorough examination should be performed in postmenopausal women, especially in those with HPV infection, with or without vaginal bleeding, vaginal discharge, palpable vaginal masses, or pain. Although the prognosis of PMMV is very poor, prompt diagnosis may improve prognosis.

## Author contributions

**Conceptualization:** Jung-Woo Park.

**Data curation:** Soyoung Park, Seo-Hee Rha.

**Formal analysis:** Soyoung Park.

**Investigation:** Seo-Hee Rha.

**Methodology:** Sujin Kim.

**Project administration:** Sujin Kim, Jung-Woo Park.

**Resources:** Seo-Hee Rha.

**Supervision:** Jung-Woo Park.

**Writing – original draft:** Soyoung Park.

**Writing – review & editing:** Sujin Kim, Jung-Woo Park.
